# *Brevibacillus* DesertYSK and *Rhizobium* MAP7 stimulate the growth and pigmentation of *Lactuca sativa* L.

**DOI:** 10.1186/s43141-023-00465-1

**Published:** 2023-02-13

**Authors:** Amr M. Mowafy, Sherouk Khalifa, Ashraf Elsayed

**Affiliations:** 1 Botany Department, Faculty of Science, Mansoura University, Mansoura, 35516 Egypt; 2 Department of Biological Sciences, Faculty of Science, New Mansoura University, New Mansoura City, Egypt

**Keywords:** *Brevibacillus*, Lettuce, Phytohormones, *Rhizobium*, Siderophores

## Abstract

**Background:**

Applying microbial biostimulants during crop cultivation allows for higher sustainability levels. It reduces the need for fertilizers and environmental contaminants while enhancing plant quality. This study assessed 13 endophytic bacteria, 4 newly isolated, and 9 donated, for plant growth-promoting capabilities. Quantitative assessments of indole acetic acid (IAA), gibberellic acid (GA_3_), siderophores, ammonia, exopolysaccharides, volatile HCN, and phosphate solubilization, along with Bray–Curtis cluster analyses were performed.

**Results:**

Upon the results  we selected *Rhizobium*MAP7, *Brevibacillus* DesertYSK, *Pseudomonas* MAP8, *Bacillus*MAP3, *Brevibacillus* MAP, and *Bacillus* DeltaYSK to evaluate their effects on *Lactuca sativa *growth and pigmentation in a 30-day greenhouse pot experiment. Both *Brevibacillus *DesertYSK and *Rhizobium *MAP7surpassed other strains in growth promotional effects. They doubled shoot length (12 and 12.3 cm, respectively, when compared with 7 cm for control after 30 days), and fresh weight (0.079 and 0.084 g, respectively, when compared with 0.045 g for control after 30 days), and increased root length by at least 3-fold when compared with control (4.5 and 3.5 cm, respectively, when compared with 1.2 cm for control after 30 days). Chlorophyll content also exhibited at least a 2-fold significant increase in response to bacterization compared with control.

**Conclusions:**

This strain superiority was consistent with the in vitro assays data that showed strains capability as IAA and GA_3_producers. Also, strains were highly capable ammonia and siderophore producers and phosphate solubilizers, providing considerable hormone and nutrient levels for *L. sativa *plantsleading to improved growth parameters and appearance. These data support the notion that nodule-based bacteria are potential plant growth-promoting bacteria (PGPB) that may be used on a wider scale rather than just for legumes.

## Background

Approximately seven billion people live on the planet and expected to reach eight billion in the coming years [1]. Increasing populations lead to a constant need to expand cultivated lands, which is inconsistent with humans’ orientation toward the industrial revolution. Also, the erosion of cultivated lands due to different factors makes this a serious issue. As a result, the usage of chemicals to increase the yield productivity was the only available solution to cover this progressive food gap, although it was not the ideal one [2]. Nevertheless, prices, availability, and environmental issues stemming from chemical fertilizers, especially nitrogen fertilizers, are the real issues facing agriculture today [3]. In the last decade, interest in plant biostimulants has rapidly escalated due to the need for eco-friendly solutions to ensure maximum crop productivity [4]. Biostimulants are neither nutrients nor pesticides, but they have a positive impact on plant growth. Seaweed extracts, protein hydrolysates, amino acids, humic acid, and microorganisms in addition are among biostimulants [5]. The intended microorganisms are characterized by being beneficial to plants, e.g., plant growth-promoting bacteria which are employed as biofertilizers, biocontrol agents, and pollutant bioremediators [6, 7]. Plant growth-promoting bacteria facilitate and enhance plant growth processes via direct and indirect mechanisms [8]. Direct stimulation involves nitrogen fixation, production of phytohormones such as auxins, cytokinins, and gibberellins, solubilization of nutrients such as phosphate, and siderophore production [9]. In contrast, indirect stimulation is mainly linked to the ability of PGPB to control phytopathogen growth and ACC (1-aminocyclo-propane-1-carboxylate)-deaminase activity that delays senescence by lowering ethylene levels [10]. Challenging abiotic stresses generated by salinity, drought, and heavy metals could be alleviated by PGPB [6, 7]. In short, PGPB helps plant cope with biotic and abiotic stresses and sustainably decrease agrochemical rates, leading to reduced greenhouse gas emissions [11, 12].

In principle, bacteria inhabit the plant rhizosphere (root surface), phyllosphere (aerial plant parts), and endosphere (internal tissues). However, studies have focused on PGPB, which competently colonize the rhizosphere [12]. The rhizosphere is a highly diverse region with different types of microbes due to root secretions, which may represent 21% of photosynthetic products, and this association is regarded as the second plant genome. In return, beneficial microbes stimulate plant growth and defense, granting it the ability to overcome different types of stress [13, 14]. The identification of rhizosphere microbiome has primarily relied on cultivation method. Using recent analytical and omic techniques, bacterial populations in the rhizosphere could be precisely described [15]. Rhizospheric or endophytic PGPB benefits the host plant with the same mechanisms referred to previously. However, the greater interaction of endophytes gives them the ability to positively affect the host’s health, growth, and response to environmental conditions [16]. In general, plant genetics and environmental conditions control bacterial endophytic colonization in plant tissues [17]. It is necessary to look for region-specific microbial strains that can be used as an inoculum to promote/enhance growth to achieve desired crop yields [9]. Nevertheless, despite recent studies documenting the benefits of PGPB as inoculants for commercial crops, their use in nonleguminous crops remains underexplored [18].

We screened endophytic bacteria isolated from different plants and tissues to identify potential biostimulants. We then studied their effects on lettuce physical growth parameters and pigmentation. Lettuce is an important leafy crop produced commercially on a large scale and widely used for its nutritional value in salads and diets [19, 20]. For this significant value, the improvement of lettuce production without excessive chemical use made PGPB a suitable candidate in this study. This aim was attained through the following steps: (1) collection and isolation of the tested bacteria, (2) in vitro quantitative assessment of plant growth-promoting criteria, and (3) applying the most promising isolates to *L. sativa* and monitor their effects on growth and pigment levels in a 30-day greenhouse study.

## Methods

Indole acetic acid (IAA), gibberellic acid (GA_3_), chrome azurol S (CAS), and hexadecyltrimethylammonium bromide (HDTMA) were obtained from Sigma-Aldrich. All other chemicals were of analytical grade.

### Isolation and identification of endophytic bacteria

During March 2018, *Lotus glaber* and *Lotus creticus* plants were collected from Mansoura University gardens (latitude 31.04120° and longitude 31.35348°) and Egypt’s north coast (latitude 31.03012° and longitude 31.36270°), respectively. Plants were gently uprooted, washed in distilled water, and the large nodules with healthy appearance were carefully detached and subjected to surface sterilization in 0.2 M HgCl_2_ in 50% ethanol for 4 min. Nodules were then washed in sterilized distilled water [21].

Under aseptic conditions, nodules were cut and contents were suspended in 5 ml sterilized distilled water. Then, 200 µl was inoculated in yeast extract mannitol solid medium (YEM) (yeast extract 1 g, mannitol 10 g, NaCl 0.1 g, K_2_PO_4_ 0.5 g, MgSO_4_ 0.2 g, CaCO_3_ 1 g, and agar 15 g per 1000 ml distilled water), the inoculated plates were incubated at 28 °C for 48–72 h. Colonies with different morphology were purified by repeated streaking. The obtained isolates were stored in 50% glycerol until required. The GeneJET Genomic DNA purification Kit (Sigma, Waltham, MA, USA) was used to extract genomic DNA from isolates. The universal primers, 27f (5′-AGAGTTTGATCCTGGCTCAG-3′) and p1492r (5′-TACGGCTACCTTGTTACGACT-3′),designed to amplify a part of 16 s rRNA gene, along with the template genomic DNA were added to a 20-µl polymerase chain reaction mixture. Thermal cycling was conducted with an initial denaturation step at 95 °C for 10 min, followed by 35 cycles of 95 °C for 30 s, 65 °C for 30 s, 72 °C for 1 min, and a final extension step at 72 °C for 10 min. The sequence of the purified PCR product was obtained by DyeEx™ 2.0 Spin Kit (Qiagen PN 63,204). These sequences were analyzed by Finch TV (version 1.4.0) software and the phylogenetic trees were generated via Sea view software using representative sequences of type strain homologues organisms those were retrieved and aligned using Ribosomal Database Project (RDB). The obtained sequences were submitted to the GenBank on NCBI.

Other isolates used in this study *Bacillus* MAP3, *Brevibacillus* MAP4, *Rhizobium* MAP7, *Pseudomonas* MAP8, *Bacillus* B2L2, *Enterobacter* E1S2, *Klebsiella* MK2R2, and *Rhizobium leguminosarum RTR1001* were generously donated from Mona Agha and Marwa Magdy, Botany Department, Faculty of Science, Mansoura University [22, 23]. All the isolates were routinely cultured on LB broth media and incubated at 28 ºϹ for 48 h [24]. All the isolates used in this study and their sources are indicated in Table 2.

### In vitro screening of plant growth promoting criteria

#### Production of indole acetic acid (IAA)

Indole acetic acid production by bacterial isolates was assayed using Salkowski reagent [25]. Isolates were inoculated into 125 ml yeast extract mannitol (YEM) broth supplemented with 0.1% tryptophan and incubated for 5 days at 28 °C and 150 rpm. After cultures were centrifuged at 10,000 rpm for 10 min, 1 ml supernatant was mixed with 1 ml Salkowski reagent (2 ml 0.5 M FeCl_3_, 49 ml water, and 49 ml 70% perchloric acid) and incubated for 20 min at room temperature. Pink color intensity was measured at 530 nm on a Jenway 7315 UV–VIS spectrophotometer. Authentic IAA was used to construct a standard curve (5–100 µg/ml) to calculate IAA concentrations in samples.

### Production of Gibberellic acid (GA_3_)

For GA_3_ production, 50 ml Luria Bertani (LB) broth media (peptone 1 g, NaCl 1 g, yeast extract 0.5 g, per 100 ml distilled water) was inoculated with isolates and incubated for 2 days at 28 °C and 150 rpm. After cultures were centrifuged at 10,000 rpm for 10 min, 15 ml supernatant was mixed with 2 ml zinc acetate reagent (21.9 g zinc acetate and 1 ml glacial acetic acid in 100 ml distilled water), and the tubes were centrifuged at 2000 rpm for 15 min. Then, 5 ml supernatant was mixed with 5 ml 30% HCl and incubated at 25 °C for 75 min. The optical density at 254 nm was measured, and a GA_3_ stock solution was used to calculate GA_3_ levels in samples [26].

#### Production of HCN

King’s B solid media (peptone 20 g, MgSO_4_.7H_2_O 1.5 g, K_3_PO_4_.3H_2_O 1.8 g, agar 15 g per 1000 ml distilled water) supplemented with 0.44% glycine was used to assess volatile HCN production. Sterilized filter papers saturated with picric acid solution (2.5 g picric acid and 12.5 g Na_2_CO_3_ in 1000 ml distilled water) were placed on the upper lid of a streaked petri dish, then tightly sealed with para-film, and incubated at 28 °C for 2 days. A color change from yellow to brown indicated a positive result. The filter papers were then cut into small pieces and soaked in 2 ml distilled water to extract HCN, and color intensity was measured at 510 nm. HCN concentrations were calculated in parts per million (ppm) using the following equation [27].

Total cyanide content (ppm) = 396 × A510 nm.

#### Production of ammonia

Ammonia production was assessed in water peptone broth media (peptone 10 g, NaCl 5 g per 1000 ml distilled water). After incubation for 4 days at 30 °C and 150 rpm, 1 ml Nessler’s reagent (50 g potassium iodide, 35 ml saturated mercuric chloride, 25 ml distilled water, and 400 ml 40% potassium hydroxide) was mixed with 1 ml of the obtained culture supernatant. A yellow to brown color formation indicated a positive result, and color intensity was measured at 450 nm. Ammonium sulfate was used to construct a standard curve to calculate ammonia levels in samples [28].

#### Production of exopolysaccharides (EPS)

Exopolysaccharides production broth media (sucrose 50 g, peptone 0.6 g, yeast extract 0.4 g, K_2_HPO_4_: 5.0 g, MgSO_4_.7H_2_O 0.4 g, NaCl 1.0 g per 1000 ml distilled water) was inoculated with isolates and incubated for 7 days at 28 °C and 150 rpm. After cultures were centrifuged at 6000 rpm for 15 min, a triple volume of cold acetone was added to supernatants to precipitate EPS and left overnight at 4 °C. After centrifugation at 6000 rpm for 10 min, EPS precipitates were dissolved in distilled water, then 0.5 ml was mixed with 0.5 ml 6% phenol reagent, and 2.5 ml concentrated sulfuric acid was immediately added. Color development was measured at 481 nm. A glucose standard curve was used to calculate EPS concentrations [29].

#### Nitrogen fixation assay

Jensen nitrogen-free solid media (sucrose 20 g, FeSO_4_ 0.1 g, K_2_PO_4_ 1 g, MgSO_4_ 0.5 g, NaCl 0.5 g, CaCO_3_ 2 g, Na_2_MoO_4_ 0.005 g, agar 15 g per 1000 ml distilled water) was used to assess nitrogen fixation ability of isolates. Growth is regarded as a positive sign [30]. Plates were incubated for 2 days at 28 °C.

#### Production of siderophores

Chrome azurol S. broth media (15 ml) was inoculated with isolates to assess siderophore production. Cultures were incubated for 2 days at 28 °C and 150 rpm. After culture centrifugation at 6000 rpm for 10 min, 0.5 ml supernatant was mixed with 0.5 ml CAS reagent, and the developed color was measured at 630 nm. Siderophore units were calculated according to the following formula [31]:

$$\mathrm{Siderophores units }(\mathrm{\%})= \frac{Ar-As}{Ar}x 100$$

where Ar = Absorbance of reference at 630 nm (CAS reagent only) and As = Absorbance of the sample at 630 nm.

#### Phosphate solubilization assay

The ability of isolates to solubilize phosphate was assayed by culturing in Reyes basal broth supplemented with 30 mM ferric phosphate as the insoluble phosphate source. Cultures were incubated at 28 °C for 5 days at 150 rpm. After this, 1 ml of freshly prepared reagent (125 ml sulfuric acid (5 M), 37.5 ml ammonium molybdate (0.2 M), 75 ml ascorbic acid (0.1 M), and 12.5 ml potassium antimony tartrate solution (0.274 g/100 ml)) were mixed with 5 ml supernatant. A purple to blue color formation indicated a positive result, and color was measured at 882 nm [32]. Potassium dihydrogen phosphate was used as a standard phosphate source.

#### Seed biopriming assay

Two methods are used to estimate 1-aminocyclo-propane-1-carboxylate-deaminase (ACC-deaminase) activity; directly by quantitatively estimating enzyme activity or indirectly using a germinating seed bioassay (used here). The bioassay was conducted on six potential bacterial isolates based on the data from in vitro assays and concluded by Bray–Curtis cluster analysis. *Vigna unguiculata* (Giza 716) and *Hordeum vulgare* (Giza 137) seeds were surface sterilized with 20% NaOHCl for 3 min and washed in deionized water. Then, seeds were germinated on 1% water agar medium for 48 h. The germinated seeds were further soaked in 20 ml isolate cultures for 1 h and then placed in Petri dishes containing wet filter paper and incubated in dark at 30 °C. Seedling growth parameters were calculated after 3 days. Vigor indices I and II were calculated according to the following equations [33].

$$\mathrm{Vigor}\;\mathrm{index}\;\mathrm I\;=\;\mathrm{Germination}\%\;\times\;\mathrm{Seedling}\;\mathrm{length}\;(\mathrm{cm})$$

$$\mathrm{Vigor}\;\mathrm{index}\;\mathrm{II}\;=\;\mathrm{Germination}\%\;\times\;\mathrm{Seedling}\;\mathrm{weight}\;(\mathrm g/\mathrm{plant})$$

#### High performance liquid chromatography (HPLC) analysis of IAA and GA_3_ production by selected isolates

Culture supernatants of the six isolates cultivated in YEM broth supplemented with 0.1% tryptophan and LB broth were used for IAA and GA_3_ estimation respectively by HPLC. After shifting the pH to 2.8 using HCl, equal volumes of ethyl acetate were added three times to extract hormones [34]. After evaporation of ethyl acetate fraction at room temperature, residues were dissolved in 500 µl pure methanol and analyzed by HPLC (Chemito 6600 Isocratic) using an ultraviolet (UV) detector and a 5-µm reverse-phase Supelcosil C18 column (39 × 300 mm). UV detector wavelengths were 280 nm and 208 nm for IAA and GA_3_, respectively. Hormones were quantified using corresponding peak areas of authentic IAA and GA_3_ standards.

### Greenhouse pot experiment on Lactuca sativa L. seeds

After identifying the most promising six isolates from the aforementioned assays, a pot study was designed to test the effects of isolates on a set of morphological and physiological parameters in lettuce plants. A pure seed strain was obtained from the Faculty of Agriculture, Mansoura, Egypt and a homogeneous lot of them were selected. Triplicate pots (30 seeds/pot) each containing 250 g sterilized peat moss soil were prepared for each treatment. Before cultivation, the soil was sterilized in customized sterilization plastic bags at 121 °C and 1.5 atm for 20 min. Control pots were supplied with the same soil without bacterial inoculation. Peat moss soil characteristics are shown (Table 1).

**Table 1:** Chemical properties of the used peat moss soil in the pot experiment of *Lactuca sativa*, L

Parameter	Value
Organic carbon (%)	56.42
Electric conductivity (EC (ds.m^−1^))	1.13
pH	3.9
Total Nitrogen (%)	0.97
Total phosphorus (%)	0.03
Total potassium (%)	0.04
Iron (ppm)	876
Zinc (ppm)	105.3
Water-holding capacity (WHC)	453.12

*Lactuca sativa* L. seeds were surface sterilized using 0.01% HgCl_2_ solution for 3 min. The bacterial solution used for irrigation was prepared as follows. Isolates (10^7^ colony forming units ml^−1^) were inoculated into LB broth and incubated for 24–48 h (the incubation period depends on the strain) at 28 °C to get *A*_600_ = 0.5. After centrifugation at 6000 rpm for 10 min, the cell mass was washed in distilled water and collected again by centrifugation. The pellet was finally resuspended in irrigation tap water (*A*_600_ = 0.5) and 100 ml was applied for each pot. Irrigation process with bacterial solutions was reapplied one week after the first irrigation. Control plants were irrigated throughout the study with equal volumes of tap water only. The study was conducted in the greenhouse of the Faculty of Science, Mansoura University, in normal field conditions of humidity, temperature, light, and day/night patterns. Samples (10 samples/treatment at each time point) were collected on the 9th, 15th, 20th, and 30th day after study commencement and were used to assess growth parameters (shoot length, root length, fresh and dry weight for shoots and roots, and the number of leaves) and pigment levels (chlorophyll a, chlorophyll b, and carotenoids).

The dimethyl sulfoxide (DMSO) method was used to extract pigments; 1 ml DMSO was added to 0.1 g plant leaves at 65 °C for 20 min [35]. Plant photosynthetic pigments (chlorophyll a, chlorophyll b, and carotenoids) were determined at all stages of plant growth using spectrophotometric methods described for chlorophylls [36], carotenoids [37], and total chlorophylls [38]. Pigment fraction concentrations were calculated as μg/ml using the following equations.

$$\mathrm{Chlorophyll}\;\mathrm{a}\;=\;10.3\;{\mathrm{E}_{663}}-0.918\;{\mathrm{E}_{644}}\;=\;\upmu\mathrm{g}/\mathrm{ml}$$

$$\mathrm{Chlorophyll}\;\mathrm{b}\;=\;19.7\;{\mathrm{E}_{644}}-3.87\;{\mathrm{E}_{663}}\;=\;\upmu\mathrm{g}/\mathrm{ml}{E}$$

$$\mathrm{Carotenoids}\;=\;5.02\;{\mathrm{E}_{480}}\;=\;\upmu\mathrm{g}/\mathrm{ml}$$

$$\mathrm{Total}\;\mathrm{chlorophylls}\;=\;7.04\;{\mathrm{E}_{645}}\;+\;20.27\;{\mathrm{E}_{663}}\;=\;\upmu\mathrm{g}/\mathrm{ml}$$

### Data analysis

A Bray–Curtis cluster analysis was performed to group isolates based on their efficiency to choose the most promising isolates for the lettuce study. Analyses were performed using Biodiversity Pro 2 software 2016.

For biological replicates (seed growth parameters and *L. sativa* plants), measures were taken from 10 replications in a completely randomized design. For technical replicates (plant growth-promoting criteria and pigment content), three samples were measured. Data were subjected to one-way analysis of variance (ANOVA). This was followed by Duncan’s test with a probability level *P* ≤ 0.05 using the COSTAT software program.

## Results

### Molecular identification of endophytic PGPB

Four bacterial isolates, DeltaYSK, DesertYSK, DeltaPSK, and DesertPSK were obtained from the root nodules of *L. glaber* and *L. creticus* plants (Table 2) and were identified using 16S rRNA gene sequence analysis. DeltaYSK showed a high level of sequence identity (99.2%) with *Bacillus flexus* and was named *Bacillus* DeltaYSK (Accession number; MT012831). DesertYSK showed a high sequence similarity (99.28%) with *Brevibacillus parabrevis* and was named *Brevibacillus* DesertYSK (Accession number; MT012893). The isolates, DeltaPSK and DesertPSK showed high levels of sequence identity (99.7% and 99.85%, respectively) with *Enterobacter cloacae* LMG 2683 and *Enterobacter cloacae* ATCC 23,373, respectively, so they were given the names *Enterobacter DeltaPSK* (Accession number; MT012829) and *Enterobacter* DesertPSK (Accession number; MT012825), respectively. Table 2 shows all the strains used in this study.

**Table 2:** The 13 isolates used in this study and their accession number in Genbank and isolation sources

Isolates	Accession number	Source/reference
*Brevibacillus* MAP4	MG214652	Nodules of *Phaseolus vulgaris* [22]
*Bacillus* MAP3	MG214652	Nodules of *Phaseolus vulgaris* [22]
*Pseudomonas* MAP5	MG214654	Nodules of *Phaseolus vulgaris* [22]
*Rhizobium* MAP7	MG214656	Nodules of *Phaseolus vulgaris* [22]
*Pseudomonas* MAP8	MG214655	Nodules of *Phaseolus vulgaris* [22]
*Rhizobium* RTR1001	EMCC No.1130	Egypt Microbial culture collection “MIRCEN”
*Bacillus* DeltaYSK	MT012831	Nodules of *Lotus glaber*, this study
*Brevibacillus* DesertYSK	MT012893	Nodules of *Lotus creticus,* this study
*Enterobacter* DeltaPSK	MT012829	Nodules of *Lotus glaber*, this study
*Enterobacter* DesertPSK	MT012825	Nodules of *Lotus creticus*, this study
*Bacillus* B2**L**2	MK574870	Leaf of *Triticum vulgare*, [23]
*Enterobacter* E1**S**2	MK574871	Stem of *Triticum vulgare*, [23]
*Klebsiella* MK2**R**2	MK464251	Root of *Phragmites australis*, [23]

### In vitro* screening of plant growth-promoting traits*

#### ***Production of IAA and GA***_***s***_

Our data indicated that all the thirteen isolates produced IAA, but they differed significantly in their ability to produce this phytohormone (Table 3). The highest IAA (130.34 µg/ml) levels were generated by *Bacillus* DeltaYSK, followed by *Brevibacillus* DesertYSK (126.97 µg/ml). GA_3_ produced by *Brevibacillus* DesertYSK was significantly higher than all other isolates (385 µg/ml), followed by the *Rhizobium* MAP7 isolate (207 µg/ml).

**Table 3:** Quantitative assays of isolates plant growth promoting traits including indole acetic acid (IAA), gibberellic acid (GA_s_)_,_ exopolysaccharides (EPS), hydrogen cyanide (HCN), ammonia, and siderophores production in addition to phosphate solubilization and qualitative assessment of nitrogen fixation ability

Isolates	IAA (µg/ml)	GA_s_ (µg/ml)	EPS (mg/l)	HCN (ppm)	Ammonia (mg/ml)	Solubilized phosphate (mg/l)	Siderophores unit (%)	N_2_ fixation
*Brevibacillus* MAP4	58.43 ± 0.75^ h^	160.5 ± 1.32^d^	582 ± 0.34^e^	12.63 ± 0.01^d^	18.8 ± 0.01^ l^	45.8 ± 0.26^d^	50.61 ± 1.6^b^	+
*Bacillus* MAP3	74.16 ± 0.31^d^	140.5 ± 0.28^ h^	153.8 ± 0.62^ h^	10.68 ± 0.14^e^	32.8 ± 0.00^ g^	ND	43.6 ± 0.4^d^	ND
*Pseudomonas* MAP5	68.55 ± 0.46^f^	154.2 ± 0.25^f^	606.8 ± 0.00^a^	ND	36.2 ± 0.3^d^	ND	ND	+
*Rhizobium* MAP7	82.03 ± 0.37^c^	207 ± 1.61^b^	601.2 ± 0.1^b^	87.52 ± 0.6^a^	50.5 ± 0.47^c^	66.2 ± 1.1^a^	71.6 ± 0.00^a^	+
*Pseudomonas* MAP8	69.67 ± 1.06^e^	156.4 ± 0.40^e^	334.9 ± 0.01^ g^	13.07 ± 1.1^d^	24.8 ± 0.22^j^	36.8 ± 0.00^e^	41.17 ± 0.17^f^	+
*Rhizobium* RTR1001	64.05 ± 0.44^ g^	145.8 ± 1.15^ g^	131.5 ± 0.00^j^	ND	26.6 ± 0.83^ h^	31.5 ± 0.26^f^	39.39 ± 0.34^ h^	+
*Bacillus DeltaYSK*	130.34 ± 0.10^a^	132.4 ± 0.75^j^	602.5 ± 0.97^b^	18.31 ± 2.7^c^	32.7 ± 0.97^f^	55 ± 0.37^c^	40.29 ± 0.82^ g^	+
*Brevibacillus* DesertYSK	126.97 ± 0.19^b^	385 ± 0.49^a^	587 ± 0.48^d^	57.86 ± 0.07^b^	59.7 ± 0.00^a^	57.6 ± 0.64^b^	38.46 ± 0.21^i^	+
*Enterobacter* DeltaPSK	57.31 ± 0.75^i^	127.6 ± 0.8^ k^	152.9 ± 0.07^i^	ND	22.9 ± 0.02^ k^	ND	35.4 ± 0.00^ k^	ND
*Enterobacter* DesertPSK	35.96 ± 0.31^ k^	137.9 ± 1.42^i^	372 ± 0.11^f^	ND	33.8 ± 0.84^e^	ND	36.5 ± 0.00^j^	ND
*Bacillus* B2L2	15.74 ± 0.01^ m^	123.5 ± 0.44^ l^	ND	ND	51.7 ± 0.01^b^	ND	42 ± 1.4^e^	+
*Enterobacter* E1S2	26.97 ± 0.10^ l^	173.6 ± 0.20^c^	ND	ND	27.3 ± 0.00^ h^	ND	34 ± 0.73^ l^	ND
*Klebsiella* MK2R2	39.33 ± 0.19^j^	132.2 ± 0.70^j^	599.8 ± 0.06^c^	ND	26 ± 0.21^i^	27.3 ± 0.00^ g^	47 ± 0.22^c^	ND
LSD	0.52	0.7	0.68	1.2	0.44	0.42	0.56	−

Values are the average ± standard error (n = 3). Different letters within each column means values are significantly different at *P* ≤ *0.05*

#### Production of EPS, HCN, and ammonia

Three out of all isolates did not produce EPS. The maximum EPS levels were recorded for *Pseudomonas* MAP5, *Rhizobium* MAP7, and *Bacillus* DeltaYSK (606.8, 601.2, and 602.5 mg/l respectively). As for volatile HCN production, 6 out of 13 isolates generated positive results: *Brevibacillus* MAP4, *Bacillus* MAP3, *Rhizobium* MAP7 (87.52, the highest amount), *Pseudomonas* MAP8, *Bacillus* DeltaYSK, and *Brevibacillus* DesertYSK. All isolates produced ammonia; the highest value (59.7 mg/ml) was assayed for *Brevibacillus* DesertYSK.

#### Production of soluble phosphate and siderophores, and nitrogen fixation abilities

Eight isolates solubilized phosphate, but with different levels. *Rhizobium* MAP7, *Brevibacillus* DesertYSK, and *Bacillus* DeltaYSK exhibited the highest phosphate solubilization abilities (66.2 mg/l, 57.6 mg/l, and 55 mg/l, respectively). Only one isolate did not produce siderophores. Quantitatively, *Rhizobium* MAP7 produced the highest siderophore levels (71.6%), followed by *Brevibacillus* MAP4 (50.61%). Nitrogen fixation abilities were qualitatively assessed by growth on nitrogen-free medium; eight isolates possessed this ability (Table 3).

**Fig. 1:**
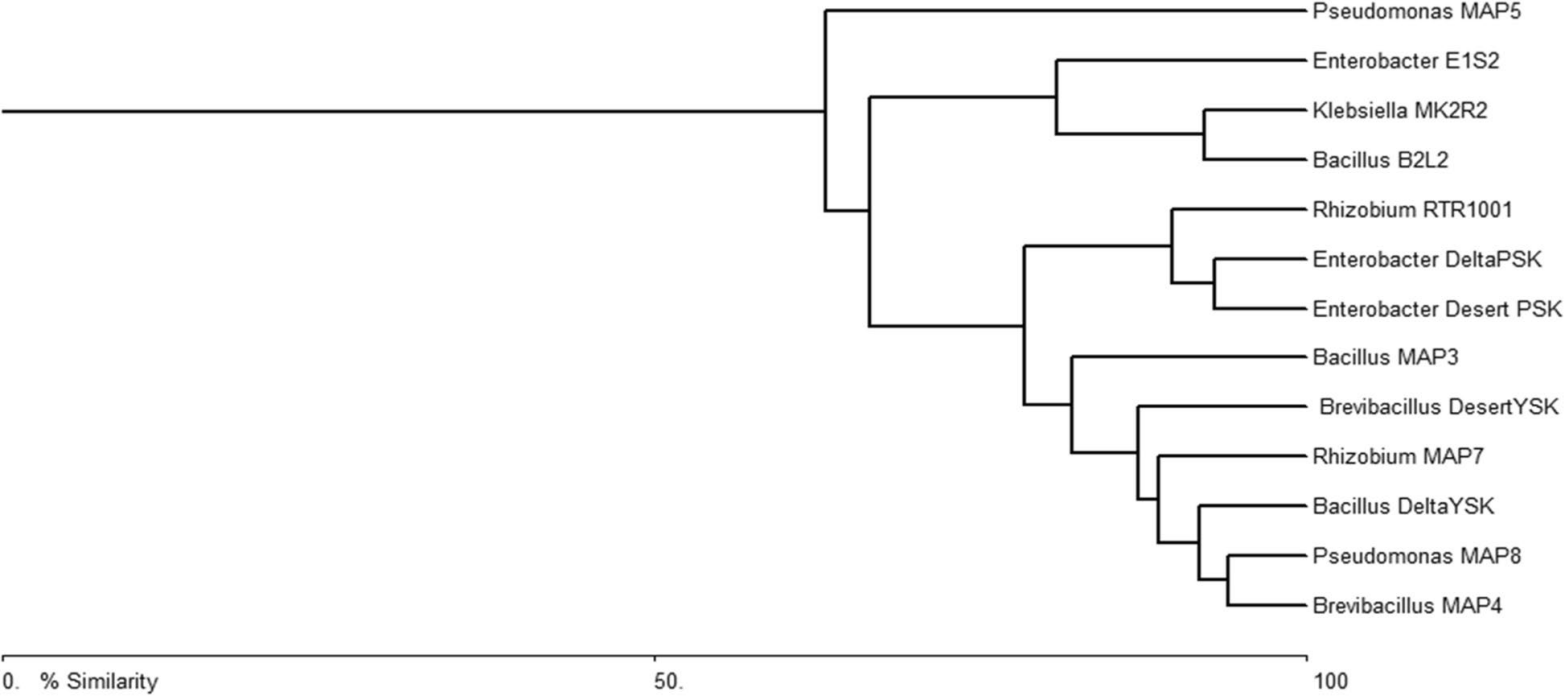
Bray–Curtis cluster analysis based on the assessed plant growth promoting criteria to choose the most promising isolates. Six isolates were clustered together according to their plant growth promoting criteria: *Rhizobium* MAP7, *Brevibacillu*s DesertYSK, *Pseudomonas* MAP8, *Bacillus* MAP3, *Brevibacillus* MAP4 and *Bacillus* DeltaYSK. This analysis was performed by Biodiversity Pro 2 software 2016

As illustrated in Fig. 1, Bray–Curtis cluster analysis bring six isolates together for their potential plant growth promoting criteria: *Rhizobium* MAP7, *Brevibacillus* DesertYSK, *Brevibacillus* MAP4, *Pseudomonas* MAP8*, Bacillus* DeltaYSK, and *Bacillus* MAP3*.*

These six strains were subjected to HPLC analysis to verify IAA and GA_3_ production levels; hormones were indeed produced, as previously corroborated by spectrophotometric analysis. However, while levels were not congruent, the highest producing strains were the same in both methods (Table 4). *Brevibacillus* DesertYSK generated the highest IAA and GA_3_ production levels in both assays. These analyses confirmed that selected strains produced phytohormones. Similarly, these methodologies showed that the spectrophotometric method for IAA and GA_3_ qualitative assessment was adequate, but quantitative HPLC measurements were more reliable.

**Table 4:** The amounts of indole acetic acid (IAA) and gibberellic acid (GA_3_) produced by *Rhizobium* MAP7, *Brevibacillus* DesertYSK, *Pseudomonas* MAP8, *Bacillus* MAP3, *Brevibacillus* MAP4 and *Bacillus* DeltaYSK based on HPLC analysis

Strains	IAA µg/ml	GA_s_ µg/ml
*Rhizobium* MAP7	0.60 ± 0.05^a^	109.40 ± 2.1^a^
*Brevibacillus* DesertYSK	1.14 ± 0.1^b^	193.80 ± 1.6 ^b^
*Pseudomonas* MAP8	0.52 ± 0.1^c^	119 ± 2.7 ^a^
*Bacillus* MAP3	0.40 ± 0.03^c^	87.20 ± 1.9 ^c^
*Brevibacillus* MAP4	0.26 ± 0.01^d^	118.20 ± 2.9 ^a^
*Bacillus* DeltaYSK	0.88 ± 0.11^a^	134.80 ± 1.5^d^

Values are the average ± standard error (*n* = 3). Different letters within each column means values are significantly different at *P* ≤ 0.05

These six isolates were used for germinating seed bioassays. They positively affected seed germination, particularly root length (at least threefold of control plants), indicating effective ACC-deaminase activity. As for *Vigna unguiculata* seedlings, *Rhizobium* MAP7 was the most effective isolate affecting root elongation, attaining a fivefold length increase compared to controls (Table 5). The highest root elongation in *Hordeum vulgare* seedlings was recorded for *Bacillus* DeltaYSK, which generated a sixfold increase in root length compared with controls (Table 6). These results suggested that *Rhizobium* MAP7 and *Bacillus* DeltaYSK generated the highest vigor indices for *Vigna unguiculata* and *Hordeum vulgare*, respectively*.*

**Table 5:** Germination parameters of *Vigna unguiculata* seedlings in response to biopriming by *Rhizobium* MAP7, *Brevibacillus* DesertYSK, *Pseudomonas* MAP8**,*** Bacillus* MAP3**,*** Brevibacillus* MAP4, and *Bacillus* DeltaYSK

Treatment	Seedling weight (g/plant)	Shoot length (cm)	Root length (cm)	Vigor index I	Vigor index II
Control	0.396 ± 0.01^*f^	1.3 ± 0.02^f^	1 ± 0.01^f^	218.5 ± 0.77^ g^	37.62 ± 0.16^ g^
*Rhizobium* MAP7	0.991 ± 0.067^a^	5.5 ± 0.0^a^	5 ± 0.007^a^	997.5 ± 0.57^a^	94.14 ± 1.6^a^
*Brevibacillus* DesertYSK	0.580 ± 0.002^d^	4.1 ± 0.11^c^	3 ± 0.0^e^	698.5 ± 0.19^c^	55.1 ± 0.23^e^
*Pseudomonas* MAP8	0.647 ± 0.008^c^	3.2 ± 0.0^e^	3.5 ± 0.01^c^	636.5 ± 0.91^f^	61.47 ± 0.22^d^
*Bacillus* MAP3	0.687 ± 0.024^c^	3.1 ± 0.06^e^	4 ± 0.05^b^	674.5 ± 0.04^e^	65.27 ± 0.02^c^
*Brevibacillus* MAP4	0.540 ± 0.00^e^	4 ± 0.13^d^	3.2 ± 0.19^d^	684 ± 0.16^d^	51.3 ± 0.04^f^
*Bacillus* DeltaYSK	0.722 ± 0.039^b^	5 ± 0.0^b^	3.3 ± 0.04^d^	788.5 ± 0.09^b^	68.59 ± 0.66^b^
LSD	0.03	0.06	0.07	0.35	0.46

Values are the average ± standard error (*n* = 10). Different letters within each column means values are significantly different at *P* ≤ 0.05

**Table 6:** Germination parameters of *Hordeum vulgare* seedlings in response to biopriming by *Rhizobium* MAP7, *Brevibacillus* DesertYSK, *Pseudomonas* MAP8, *Bacillus* MAP3*, Brevibacillus* MAP4, and *Bacillus* DeltaYSK

Treatment	Seedling weight (g/plant)	Shoot length (cm)	Root length (cm)	Vigor index I	Vigor index II
Control	0.092 ± 0.0^f^	2.1 ± 0.1^f^	0.5 ± 0.0^e^	249.6^b^ ± 0.05	8.74 ± 0.17^f^
*Rhizobium* MAP7	0.143 ± 0.07^c^	4.5 ± 0.36^c^	1.6 ± 0.1^d^	585.6^d^ ± 0.47	14.58 ± 0.28^c^
*Brevibacillus* DesertYSK	0.182 ± 0.01^b^	5.7 ± 0.4^b^	3.3 ± 0.1^a^	873.6^f^ ± 4.91	17.29 ± 3.7^b^
*Pseudomonas* MAP8	0.118 ± 0.14^d^	1.4 ± 0.0^ g^	2 ± 0.2^c^	326.4^a^ ± 0.8	11.21 ± 0.02^d^
*Bacillus* MAP3	0.138 ± 0.07^c^	2.4 ± 0.2^e^	2.2 ± 0.0^b^	441.6^c^ ± 1.67	13.11 ± 0.1^e^
*Brevibacillus* MAP4	0.119 ± 0.09^e^	3 ± 0.1^d^	1.6 ± 0.1^d^	430.2^c^ ± 0.21	11.31 ± 0.76^e^
*Bacillus* DeltaYSK	0.201 ± 0.01^a^	7 ± 0.4^a^	3.4 ± 0.2^a^	988.8^e^ ± 1.67	19.10 ± 2.1^a^
LSD	0.005	0.29	0.1	0.6	1.23

Values are the average ± standard error (*n* = 10). Different letters within each column means values are significantly different at *P* ≤ 0.05

### The effects of selected isolates on L. sativa growth

**Fig. 2:**
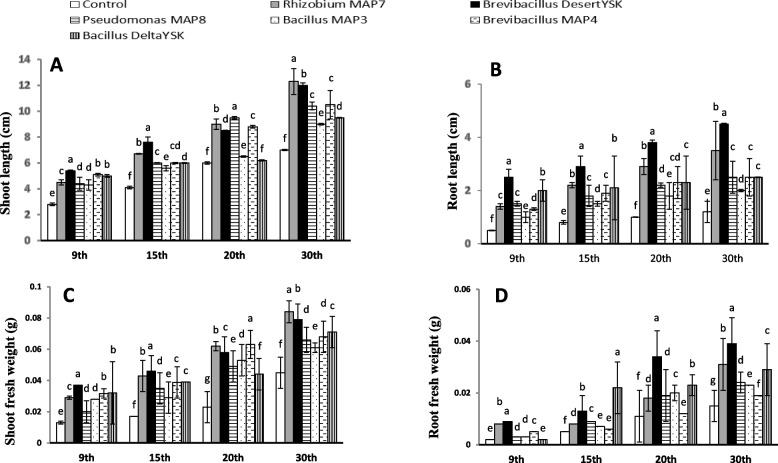
Effect of *Rhizobium* MAP7, *Brevibacillus* DesertYSK, *Pseudomonas* MAP8, *Bacillus* MAP3, *Brevibacillus* MAP4 and *Bacillus* DeltaYSK on A shoot length, B root length, C shoot fresh weigh, and D root fresh weight of *Lactuca sativa* after germination for 9, 15, 20, and 30 days. The bars represent the mean value (*n* = 10) and the error bar represents ± standard error. Different lower-case letters indicate significant differences between the treatments “at particular sampling time” according to Duncan’s test at P ≤ 0.05

Representative shoot lengths of the lettuce plant at germination and vegetative stages, indicated that seeds treated with *Rhizobium* MAP7, *Brevibacillus* DesertYSK, *Brevibacillus* MAP4, and *Pseudomonas* MAP8 showed significant length increases when compared with controls (Fig. 2A). The highest significant value was reported for *Rhizobium* MAP7 at day 30, reaching 12.3 cm compared to the 7 cm for control.

Treatment with *Brevibacillus* DesertYSK and *Rhizobium* MAP7 significantly increased root length, while *Bacillus* MAP3, *Brevibacillus* MAP4, *Pseudomonas* MAP8*,* and *Bacillus* DeltaYSK showed less significant increases compared with controls. The highest value was recorded for bacterization with *Brevibacillus* DesertYSK at all growth stages (Fig. 2B).

**Fig. 3:**
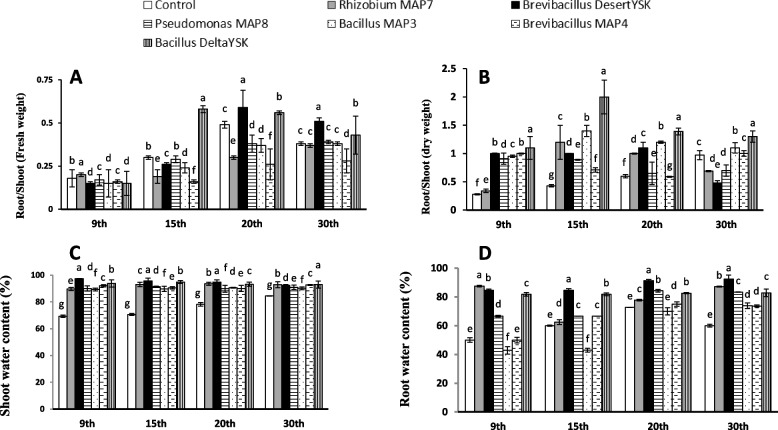
Effect of *Rhizobium* MAP7, *Brevibacillus* DesertYSK, *Pseudomonas* MAP8, *Bacillus* MAP3, *Brevibacillus* MAP4 and *Bacillus* DeltaYSK on A root/shoot fresh weight, B root/shoot dry weight, C shoot water content, and D root water content of *Lactuca sativa* after germination for 9, 15, 20, and 30 days. The bars represent the mean value (*n* = 10) and the error bar represents ± standard error. Different lower-case letters indicate significant differences between the treatments “at particular sampling time” according to Duncan’s test at P ≤ 0.05

Both shoot and root fresh weights in response to all treatments increased significantly, while bacterization with *Brevibacillus* DesertYSK and *Rhizobium* MAP7 surpassed the others in almost all growth stages (Fig. 2C, D). The alteration of fresh biomass partitioning indicated that *Brevibacillus* DesertYSK and *Bacillus* DeltaYSK are the ones that showed the difference after 20 and 30 days of growth in root/shoot fresh weight ratio. However, the dry weight root/shoot ratio significantly increased in response to all treatment after 9, 15, and 20 days of growth (Fig. 3A, B).

Although the water content increased significantly in response to all treatments, the root content of water retained at the highest level in response to *Brevibacillus* DesertYSK (Fig. 3C, D).

**Fig. 4:**
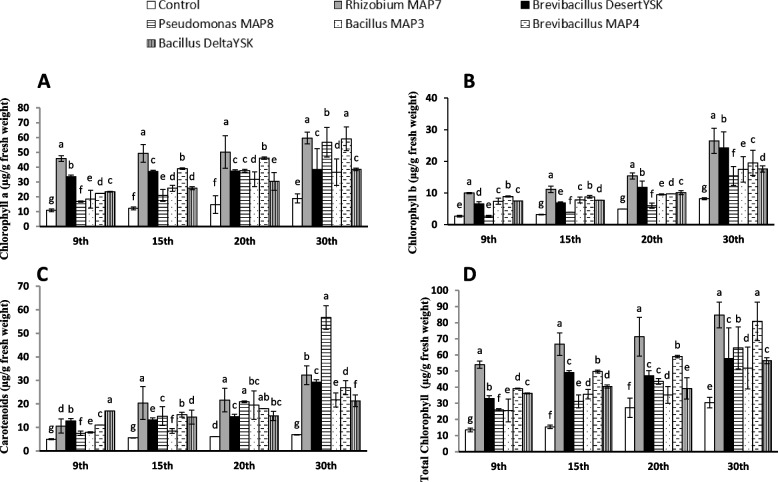
Effect of *Rhizobium* MAP7, *Brevibacillus* DesertYSK, *Pseudomonas* MAP8, *Bacillus* MAP3, *Brevibacillus* MAP4 and *Bacillus* DeltaYSK on A chlorophyll a, B chlorophyll b, C carotenoids, and D Total pigments of *Lactuca sativa* after germination for 9, 15, 20, and 30 days. The bars represent the mean value (*n* = 10) and the error bar represents ± standard error. Different lower-case letters indicate significant differences between the treatments “at particular sampling time” according to Duncan’s test at P ≤ 0.05

All treatments led to a dramatic increase in chlorophyll a compared with controls; the highest value was recorded for *Rhizobium* MAP7 bacterization (three times the control) in 30-day old seedlings (Fig. 4A). Treatment with *Rhizobium* MAP7 (three times the control) and *Brevibacillus* DesertYSK caused a significant increase in chlorophyll b compared with controls (Fig. 4B). In terms of carotenoid content, levels increased significantly by *Pseudomonas* MAP8, *Rhizobium* MAP7, and *Brevibacillus* DesertYSK treatments (Fig. 4C). The highest value was recorded on the final study day for *Pseudomonas* MAP8 treatment (56.7 µg/g fresh weight). The response of total chlorophyll increase is the toll of the mentioned increases in chlorophyll a, chlorophyll b, and cartenoids in response to the used biostimulants that were in descending order as follows, *Pseudomonas* MAP8, *Rhizobium* MAP7, *Brevibacillus* MAP4, *Brevibacillus* DesertYSK, and *Bacillus* DeltaYSK*.*

**Fig. 5:**
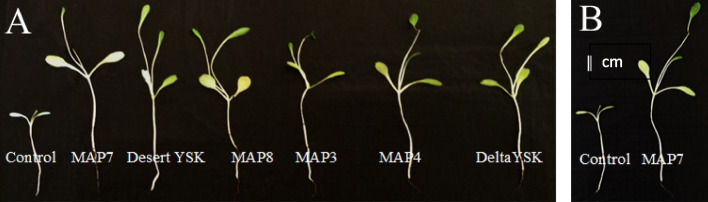
A The growth of *Lactuca sativa* seedlings on the 30th day of the greenhouse experiment after different bacterial treatments compared to the control (MAP7 = *Rhizobium *MAP7, DesertYSK = *Brevibacillus* DesertYSK, MAP8 = *Pseudomonas* MAP8, MAP3 = *Bacillus* MAP3, MAP4 = *Brevibacillus* MAP4, and DeltaYSK = Bacillus DeltaYSK), B control versus *Rhizobium* MAP7 treated plants

From our data, all treatments improved lettuce growth and surpassed controls; lettuce seedling showed greatly improved growth parameters (Fig. 5A), with evident differences in appearance between biostimulant-treated lettuce plants and controls (Fig. 5B).

## Discussion

The plant growth-promoting traits of isolates from different plant tissues showed that strains coinhabiting in root nodules with rhizobia were more efficient when compared with endophytic counterparts. This was not only evident from in vitro assays, but also seed biopriming assays*.* Based on Bray–Curtis analyses, six isolates clustered together included *Rhizobium* MAP7, *Brevibacillus* DesertYSK, *Brevibacillus* MAP4, *Pseudomonas* MAP8, *Bacillus* DeltaYSK, and *Bacillus* MAP3. All were derived from the root nodules of legumes. Our results evidently support that a lot is still unknown about the nodule-inhabiting microbes other than rhizobia, although they might be of high potentiality as a source for PGPB.

### The growth-promoting criteria of isolates

To sum up how the plant growth promotion was made possible by PGPB, here is a list of criteria to be checked. Firstly, IAA produced by rhizobacteria mainly affects root systems by increasing growth and branching total count, leading to increased soil-contact surface areas. These improvements increase the available nutritional pool and the growth potential of plants. In this study, *Brevibacillus* DesertYSK and *Bacillus* DesertYSK produced the highest IAA levels based on in vitro assays (Tables 3 and 4) and greatly promoted lettuce and barley root elongation (Table 6 and Fig. 2A), but not cowpea; here, *Rhizobium* MAP7 bacterization generated the highest root length values. This result agreed with previous research reporting IAA production from the same bacterial species isolated from a citrus plant [39, 40]. *Rhizobium* MAP7 is one of the highest IAA producers, and this might be explained by compatibility between this strain and *V. unguiculata* as a legume, although it might not be the optimum host among other legumes [41].

Gibberellin produced by PGPB affects the shoot systems of plants after upward translocation. In this study, the shoot system got on board too in the lettuce experiment. Regarding lettuce weight and water content, as *Brevibacillus* DesertYSK produced the highest GA_3_ levels (Tables 3 and 4), this may explain the enhancement in shoot growth and fresh lettuce weight by this strain after nine and 15 days of growth (Figs. 2 and 3). This strain was continuing to advance in influence, despite not being the most significant at the time of experiment termination. For *V. unguiculata*, *Rhizobium* MAP7 was a more favorable biostimulant. This result agreed with a previous report where *Acinetobacter calcoaceticus*, the producer of different GA_3_ and a phosphate solubilizer, enhanced cucumber, Chinese cabbage, and crown daisy growth [42, 43].

One of the functions of PGPB is to allow plants to use unavailable macronutrients such as nitrogen. The use of *Rhizobium* MAP7 and *Brevibacillus* DesertYSK, as symbiotic and free nitrogen fixers (Table 3), therefore enhances lettuce (Figs. 2 and 3), barley, and cowpea growth (Table 5). This result agreed with previous studies, where the positive effects of these strains on several plants were attributed to their nitrogen-fixing abilities [44–46]. Ammonia production is not an uncommon trait in endophytic bacteria from root nodules and is related to nitrogen fixation [47]. The nitrogen fixers in this study were outstanding in terms of ammonia production (Table 3).

Phosphate solubilizing bacteria enhance the availability of insoluble phosphate. *Rhizobium* MAP7, *Brevibacillus* DesertYSK, and *Bacillus* DeltaYSK were the highest phosphate solubilizing strains in this study. Relevant bacterial strains were of potential phosphate solubilizing ability that enabled them to improve tested plants’ growth [48–50]. Iron chelation by PGPB siderophores provides iron for plant growth. In this study, *Rhizobium* MAP7 was the highest siderophore producing isolate. Most recently, *Rhizobium leguminosarium* was found to produce siderophores even under kitazin stress that enable pea to grow in fungicide enriched soil [51].

Volatile cyanogen production by compatible PGPB helps alleviate biotic stressors such as weeds, phytopathogens, and abiotic stressors such as salt [52–54]. *Rhizobium* MAP7 followed by *Brevibacillus* DesertYSK showed the highest HCN levels (Table 3). A previous report indicated the ability of *Rhizobium* to produce HCN [51]. However, *Brevibacillus* was regarded as a noncyanogenic organism [55].

*Pseudomonas* MAP5 produced the highest EPS levels (Table 3) in agreement with a previous study stating that *Pseudomonas putida* produced huge EPS levels when compared with other isolates [56]. EPS generation is an important factor for plant interactions, not only for beneficial microbes but also for pathogens [57].

1-Aminocyclo-propane-1-carboxylate-deaminase activity by PGPB is a topical subject in the literature. Our data showed that *Rhizobium* MAP7 and *Bacillus* DeltaYSK generated the highest vigor indices and root and shoot lengths for *V. unguiculata* and *H. vulgare*, respectively. Thus, seeds treated with these isolates were more vigorous when compared with controls and other treatments. The increased root elongation may be attributable to the ACC-deaminase activity of these strains. The first report on ACC deaminase activity in rhizobia was in *Rhizobium leguminosarum* bv. *viciae* 128C53K [58]. Several *Bacilli* also exhibit this ACC activity [59, 60]. By serving as a sink for ACC, the cleavage of ACC by the bacterium supplying ACC deaminase reduces the quantity of ACC and subsequently ethylene leading to delayed senescence [61]. The high I and II vigor indices induced by *Rhizobium* MAP7 and *Bacillus* DeltaYSK for *V. unguiculata* and *H. vulgare*, respectively, indicated the potential effects of these isolates on seed performance.

### L. sativa responses to PGPB

Pot studies evaluated the effects of selected PGPB on lettuce growth and pigment content. On the 30th day, all treatments, especially *Brevibacillus* DesertYSK and *Rhizobium* MAP7, in the absence of soil chemical fertilizers (other than peat moss chemical composition (Table 1)), significantly surpassed control plants for all growth parameters. This indicated significant growth promotion effects by isolate treatments, as supported by in vitro studies. *Rhizobium leguminosarum* was previously used as a potential microbial biofertilizer for *L. sativa* [62]. *Rhizobium radiobacter* was described as the best biofertilizer for lettuce cultivation compared with (NPK), vermicompost (VC), and farmyard manure (FYM) [63]. Additionally, different *Pseudomonas* strains alleviated salt stress in lettuce plants [64]. To our knowledge, our study may be the first to report *Brevibacillus* as a compatible PGPB for lettuce. Similarly, *Brevibacillus brevis* was reported as a potential promoter of cotton growth [55]. Most recently, *Brevibacillus* was isolated from maize cultivated in a semi-arid region and described as potential PGPB [65].

In terms of pigmentation, plants treated with *Rhizobium* MAP7, *Brevibacillus* DesertYSK, and *Pseudomonas* MAP8 displayed higher pigment levels (chlorophyll a, chlorophyll b, and carotenoids). Increased chlorophyll in response to PGPB was reported in several studies [66, 67]. This increased pigment content may be attributed to available nitrogen and siderophores, provided by these strains in the in vitro assays (Table 3) those that allow chlorophyll biosynthesis, a conclusion has been drawn previously [68]. Regarding the aforementioned plant growth promotion criteria, it makes sense that the pretreated yield with compatible PGPB would have better quality than quantity. Several studies suggested that PGPB improved photosynthesis in plants and affected pigment content, making plants greener [69]. Similarly, photosynthate production is believed to enhance crop growth and development when treated with PGPB.

## Conclusions

PGPB potentially functions as a novel solution to address sustainable agriculture issues [70]. Carefully selecting compatible and beneficial microorganisms from highly specific environments such as root nodules could generate noticeable and substantial differences in agriculture. In this study, both *Brevibacillus* DesertYSK and *Rhizobium* MAP7 outperformed other strains in terms of in vitro plant growth promoting traits and positively affecting *L. sativa* growth and pigmentation. This result confirmed that root nodules could be a unique repository for potential plant growth promoting bacteria. These promising strains warrant more comprehensive research and widespread applications not only for lettuce but for other crops.

## Data Availability

Not applicable.

## Abbreviations

CAS: Chrome azurol S
EPS: Exopolysaccharides
GA3: Gibberellic acid
HDTMA: Hexadecyltrimethylammonium bromide
IAA: Indole acetic acid
PGPB: Plant growth promoting bacteria
